# Evaluation of Dietary Probiotic Bacteria and Processed Yeast (GroPro-Aqua) as the Alternative of Antibiotics in Juvenile Olive Flounder *Paralichthys olivaceus*

**DOI:** 10.3390/antibiotics11020129

**Published:** 2022-01-19

**Authors:** Wonsuk Choi, Mohammad Moniruzzaman, Jinho Bae, Ali Hamidoghli, Seunghan Lee, Youn-Hee Choi, Taesun Min, Sungchul C. Bai

**Affiliations:** 1 Feeds & Foods Nutrition Research Center, Pukyong National University, Busan 48547, Korea; thm622@naver.com (W.C.); bjh2921@naver.com (J.B.); ali.hamidoghli@yahoo.com (A.H.); 2 Department of Animal Biotechnology, Jeju International Animal Research Center (JIA) & Sustainable Agriculture Research Institute (SARI), Jeju National University, Jeju 63243, Korea; monir1983@jejunu.ac.kr; 3 Aquafeed Research Center, National Institute of Fisheries Science, Pohang 37517, Korea; shlee5863@naver.com; 4 Department of Marine Bio-Materials & Aquaculture, Pukyong National University, Busan 48513, Korea; unichoi@pknu.ac.kr; 5 FAO World Fisheries University Pilot Program, Busan 48547, Korea

**Keywords:** beneficial bacteria, processed yeast, alternative of antibiotics, growth, innate immunity, histomorphology, *Edwardsiella tarda*, olive flounder

## Abstract

We investigated the three probiotic bacteria and a processed yeast (GroPro-Aqua) as the replacers of antibiotics in juvenile olive flounder. A total of seven diets were used, that is, one basal or control (CON) diet; and six other diets, of which, three diets were prepared by supplementing probiotic bacteria such as *Bacillus subtilis* WB60 (BSWB60) at 1 × 10^8^ CFU/g diet, *Bacillus subtilis* SJ10 (BSSJ10) at 1 × 10^8^ CFU/g diet, and *Enterococcus faecium* SH30 (EFSH30) at 1 × 10^7^ CFU/g diet; one diet with processed yeast (GRO) at 0.35% diet; and two other diets were supplemented with oxytetracycline (OTC) and amoxicillin (AMO) at 4 g/kg of each. Triplicate groups of fish (average 12.1 g) were fed one of the diets for eight weeks. At the end of the feeding trial, the fish that were fed the probiotic bacteria-supplemented diets had a significantly higher final weight, weight gain, and specific growth rate compared to the CON, OTC, and AMO diets. Fish that were fed the GRO diet had significantly higher feed efficiency and protein efficiency ratios than those of the fish that were fed the CON diet. Serum glutamic pyruvic transaminase, glutamic oxaloacetic transaminase, glucose, and total protein were not affected by the diets. Lysozyme activity in fish that were fed the BSSJ10, BSWB60, and EFSH30 diets were significantly higher compared to the CON and OTC diets, whereas myeloperoxidase activity of fish fed the BSWB60 and EFSH30 diets were significantly higher than those of fish fed the CON and AMO diets. Flounder growth hormone gene expressions of fish that were fed BSWB60 and GRO diets were significantly higher compared to the CON, OTC, and AMO diets. The interleukin-1β gene expression of fish that were fed the BSSJ10, BSWB60, EFSH30, OTC, and GRO diets was significantly higher than those of fish fed the CON diet. The interleukin-10 gene expression of fish that were fed the BSSJ10, EFSH30, and GRO diets was significantly higher than those of fish fed the CON and AMO diets. Posterior intestinal histology of fish showed significantly higher villi length in fish that were fed the BSSJ10, BSWB60, EFSH30, and GRO diets compared to the CON diet. After 15 days of challenge test with pathogenic bacteria *Edwardsiella tarda*, the cumulative survival rate of fish that were fed the BSSJ10, BSWB60, EFSH30, and GRO diets were significantly higher than those of fish that were fed the CON diet. Overall, the results indicate that dietary supplementation of *B. subtilis* (10^8^ CFU/g diet), *E. faecium* (10^7^ CFU/g diet), and processed yeast (GroPro-Aqua at 0.35% diet) could replace the antibiotics in terms of improving growth, immunity, gut health, and disease resistance in juvenile olive flounder.

## 1. Introduction

Aquaculture is a rapidly advancing and important source of fish protein which occurs through the rearing of fish and crustaceans in fresh, coastal, or brackish water as well as marine environments [1]. It is expected that this fast-growing industry will provide the requirements for healthy protein to keep up with the enormous global population growth to 9.5 billion that is anticipated by 2050 [2]. The growing market demand has forced the industry to maximize its production by moving towards intensive aquaculture. For the sustainable development of aquaculture, it is of utmost importance to go for intensification in aquaculture which can mitigate the problem of high protein demand. Intensive aquaculture usually needs different inputs such as feeds, antibiotics, disinfectants, insecticides, probiotics, and prebiotics [3]. However, intensive culture of fish also causes economic loss due to stress and disease outbreaks which are considered as the main drawbacks for successful aquaculture [4]. As a response to disease outbreaks in aquaculture, antibiotics are generally used in the diets as additives or in the form of a liquid mixture to eradicate the pathogens in the aquafarms as well as to increase the fish growth [5]. The excessive use of antibiotics such as oxytetracycline (OTC) and amoxicillin (AMO) can cause major problems for the sustainable development of aquaculture [5]. The overuse of antibiotics in aquaculture can facilitate the emergence of resistant bacterial strains, accumulate in fish flesh, and pollute the aquaculture environment which, ultimately, can cause public health hazards [5,6,7,8]. Due to the indiscriminate use of antibiotics as the antimicrobial growth promoters (AGPs) in animal feeds, the European Union and the Food and Drug Administration (FDA) of the USA were banned or restricted in some cases on the use antibiotics for the improvement of growth and remedial uses in disease outbreaks [9]. Recently, some European countries, especially Norway, has decreased the use of antibiotic materials in aquafarms and enhanced the use of vaccine and good aquaculture practices (GAP) as the preventive measures to control disease outbreaks in fish [5]. Nevertheless, farmers in Asian countries are still using these illegal chemicals to treat their aquaculture species where more than 80% of global aquaculture production comes from [3,7]. In a recent article, Lulijwa et al. [3] reported that there are 15 global aquaculture-producing countries, including Rep. of Korea, that are using antibiotics in aquaculture. Moreover, Choi et al. [5] found that most of the antibiotics that are used in Rep. of Korea for olive flounder aquaculture will eventually run-off to the surrounding seawater environment which may create hazardous issues in aquatic environment through the residual effects of the antibiotics. Therefore, it is badly needed to find alternatives for antibiotics for the treatment of bacterial diseases in aquafarms to obtain healthy fish and to ensure safe fish production which will not affect the public health and the aquatic environment.

Feed additives generally encompass the ingredients that are supplemented in the diets to mitigate the nutrient requirements in terms of improving the growth performance and the health status of fish [10,11,12,13]. The use of probiotics in fish feed as additives could be a better option to increase disease resistance and improve the immune status in animals compared to the excessive use of antibiotics with hazardous effects on the environment [6,14]. Probiotics can be used as a live or killed microorganisms that are supplemented in the diet or in the aquatic environment to enhance the fish performance, immune health status, as well as the water quality of the culture environment [15,16]. Several studies reported that probiotic bacteria can increase growth in terms of weight gain, specific growth rate (SGR), feed utilization like feed efficiency (FE), protein efficiency ratio (PER), as well as somatic indices such as hepatosomatic index (HSI), viscerosomatic index (VSI), and condition factor (CF) in fish and shrimp species [16,17,18,19,20,21,22,23,24,25,26]. The efficacy of bacteria as probiotics can be assessed by understanding the serum biochemical composition of blood which reflects the health status of fish. A significant increase in serum biochemical parameters such as glutamic pyruvic transaminase (GPT) and glutamic oxaloacetic transaminase (GOT) levels can cause problems in the liver which is known as the largest digestive gland in the animal. Furthermore, higher glucose (GLU) or total protein (TP) including albumin and globulin levels in blood may represent the stress condition of the animal. Therefore, it is of utmost importance to know whether the dietary bacteria have an adverse effect or not based on the serological composition in fish. Many researchers have found an unaltered serum biochemical composition in fish after oral administration of probiotic bacteria which was attributed to the beneficial effects of the bacteria [16,17,18,19,20,21,22,23,24,25]. Non-specific immune responses in terms of superoxide dismutase (SOD), lysozyme, and myeloperoxidase (MPO) are important enzymes to determine innate immunity in fish [17]. The enzymes are responsible for the regulation of reactive oxygen species (ROS) during the stress response in the animal. It has been reported that the dietary supplementation of probiotics can improve the innate immunity in fish through the regulation of ROS by SOD, lysozyme, and MPO activities [17]. Furthermore, the cellular inflammation in fish can easily be detected through the determination of some immune-related gene expressions; pro-inflammatory cytokines such as tumor necrosis factor-alpha (TNF-α), interleukin-1 beta (IL-1β), and interleukin-6 (IL-6); as well as anti-inflammatory cytokine like interleukin-10 (IL-10) which are widely studied mRNA gene expressions in fish [18]. The dietary supplementation of probiotic bacteria in fish was found to increase the cytokine gene expressions such as IL-1β, IL-6, and IL-10 as well as TNF-α which endorsed the anti-inflammatory capacity of probiotics in fish [18,19,20]. The beneficial effect of probiotic bacteria can be exerted in the gastrointestinal tract (GIT) in terms of increased digestion and assimilation of nutrients, as well as prevention or reduction of pathogenic bacterial contents in the GIT. The probiotic bacteria can balance the gut microbiota in the GIT through the homeostasis process and improve the gut environment [17,21]. The *Bacillus subtilis* is a widely used Gram-positive bacterium which has a broad spectrum of antimicrobial effects [21,22,23,24,25,26], is highly capable of enzyme production in the GIT [22,23], and the potential to increase aquaculture profitability [21]. As a beneficial bacterium, *B. subtilis* SJ-10 can significantly improve the fish performance, feed utilization, non-specific immune responses, and stress resistance in juvenile olive flounder that are fed a low fish meal diet [18,19,20]. In our previous studies, we reported on the beneficial effects of *B. subtilis* WB60 alone or combination with other probiotics in terms of improved growth, immunity, gut architecture, and stress resistance in Japanese eel [17], Nile tilapia [25], and whiteleg shrimp [26]. Likewise, Chen et al. [27,28] reported the positive effects of *B. subtilis* with multi-strains of probiotics in the diets of freshwater grass carp fish and whiteleg shrimp. Furthermore, the *Enterococcus faecium* is a Gram-positive, alpha-hemolytic or non-hemolytic bacterium which has a potential probiotic effect in aquaculture, as previously described by Da Costa Sousa et al. [29]. The researchers found that the dietary supplementation of *E. faecium* has probiotic effects and can be used at 1 × 10^8^ CFU/g to improve growth, hematology, gut microbiota, and reduce parasitic infestation in Pirarucu fish.

The dietary supplementation of yeast as single-celled microorganisms can act as a potential functional feed additive that constitutes a good source of protein, nucleic acids and nucleotides, vitamins, minerals, β-glucan, and mannan-oligosaccharides which could enhance the growth, feed palatability, as well as the innate and adaptive immune responses in animals [14,30,31,32]. Previous research has reported that yeast can play a potential role in the rate of growth, while also modulating the immune system and disease resistance in fish and crustaceans [33,34]. It is also reported that dietary yeast can modulate the pro-inflammatory cytokine gene expressions and improve the gut health in fish [35,36,37].

Olive flounder (*Paralichthys olivaceus*) is a highly demanded and potential aquaculture fish species in East Asia, especially in Japan, Rep. of Korea, and China. In Rep. of Korea in 2005, olive flounder farms were affected by huge mortality in the growing stage due to the high density culture of fish along with water quality deterioration and disease outbreaks [38]. In particular, the reasons for the great reduction of production capacity of olive founder farms were found to be due to the viral and bacterial infections in fish between 2009 and 2017 [18]. To encourage farmers to avoid using high amounts of antibiotics, alternative strategies are needed. Therefore, in this study, we investigate the dietary effects of *Bacillus subtilis* (two strains), *Enterococcus faecium,* and a commercial processed yeast (GroPro-Aqua) in olive flounder and compare them with two main antibiotics (oxytetracycline and amoxicillin) that are used in aquaculture. The effects of these feed additives were evaluated based on growth, hematology, non-specific immune responses, growth hormone, as well as pro-inflammatory and anti-inflammatory cytokine gene expressions, histomorphology, and survival against the pathogenic bacteria *Edwardsiella tarda* which is the causative agent of the disease, Edwardsiellosis.

## 2. Materials and Methods

### 2.1. Ethics Statement

The experiment was followed under the guidelines of Institutional Animal Care and Use Committee Regulations, No. 554, issued by the Pukyong National University, Busan, Rep. of Korea. Every effort was taken to minimize fish suffering.

### 2.2. Collection and Preparation of Probiotic Bacteria

The strain, *B. subtills* WB60 was extracted from the anterior intestine of Japanese eel (5 fish) that were in good condition, [17] and the bacterial strain was identified by sequential cluster analysis with 16S rDNA gene [39]. The *B. subtills* WB60 was then transferred to an incubator at 30 °C for 72 h in Luria-Bertani (LB) broth (Sigma-Aldrich, St. Louis, MO, USA) and measured at 600 nm optical density (OD600) using a UV/VIS spectrometer (Lambda 35; Perkin-Elmer, Waltham, MA, USA) according to Lee et al. [17]. In addition, *B. subtilis* SJ-10 was isolated from a Korean traditional fermented fish (5 fish), *Jeotgal* [18] and the probiotic bacteria was inoculated on LB agar at single colony and then the bacteria was transferred to a production medium at 37 °C with incubation for 16 h in a vibrating incubator (HB 201SF; Hanbaek Scientific Co., Seoul, Rep. of Korea). On the other hand, *E. faecium* was collected from the anterior intestine of healthy Nile tilapia (7 fish) and identified as described previously [29]. Briefly, the *E. faecium* was grown in de Man, Rogosa & Sharpe (MRS) broth at 36 °C for 48 h [40]. The viable number of bacterial colonies in 1 mL of aliquots was also counted using a colony counter (Suntex automatic colony counter, Taiwan) on the solid medium for establishing the growth curve of the bacterial colonies. All the live probiotic bacterial colonies were cleaned with sterile saline water and the amounts of the bacterial cells in sterile solution was adjusted at 1 × 10^8^, 1 × 10^8^ and 1 × 10^7^ CFU (colony forming unit)/g diets for *B. subtilis* WB60 [17], *B. subtilis* SJ-10 based on [18], and *E. faecium* SH30 [29], respectively.

### 2.3. Experimental Diets

The basal or control diet formulation is shown in Table 1. The fish meal (68.75% CP) from anchovy and soybean meal (47.04% CP) were incorporated in the diets as principal protein sources, whereas dietary lipid was sourced from fish oil. In addition to the control or basal diet, six feed additives were supplemented in the iso-nitrogenous and iso-lipidic experimental diets and were designated as BSWB60 (*B. subtilis* WB60 at 1 × 10^8^ CFU/g diet based on Lee et al. [17]), BSSJ10 (*B. subtilis* SJ-10 at 1 × 10^8^ CFU/g diet Hasan et al. [18]), EFSH30 (*E. faecium* SH30 at 1 × 10^7^ CFU/g diet based on Da Costa Sousa [29]), GRO (GroPro-Aqua at 0.35% diet), OTC (oxytetracycline at 4 g/kg diet), and AMO (amoxicillin at 4 g/kg diet). The GroPro-Aqua powder is a yeast-derived protein and nucleic acid-based commercial feed additive (GroPro Aqua, Angel Yeast Co. Ltd., Hubei, China) which is used as a growth and palatability enhancer in aquaculture. The GroPro-Aqua (GRO) that was supplemented in the control diet was based on Bae et al. [13] and manufacturers’ recommendation. The antibiotics, OTC and AMO were purchased from a commercial source (Samyang anipharm Co. Ltd., Seoul, Rep. of Korea) and supplemented in the control diets (4 g/kg diet of each) based on Park et al. [22] and manufacturers’ instructions. The feed formulation, diet manufacturing, and storage process was based on Bai and Kim [41]. Briefly, the ingredients in a powdered form were mixed together using a mixing machine (HYVM-1214, Hanyoung Food Machinery Co., Ltd., Gyeonggi-do, Rep. of Korea). Then, the fish oil was slowly added in the mixer with addition of approximately 30% filtered tap water to make a feed dough. The pellets of the respective diets were produced by moving the doughs inside a metallic pelleting machine (SFD-GT, Shinsung E&G, Gyeonggi-do, Rep. of Korea) without heating using a 0.2 cm diameter pore size module. The manufactured diets were then kept at standard temperature (25 °C) and dried for 48 h. The dried pellets were broken up, sieved using metallic sieving nets to make uniform-sized pellets, and stored in plastic zipper bags with tagging according to the name of the diets at −20 °C in a freezer until use for the experiment. 

### 2.4. Animals and Experimental Set-Up

Juvenile Olive flounder were obtained from a privately-owned aquaculture farm (Chungcheongnam-do, Taean-gun, Rep. of Korea). Prior to the starting of the dietary administration trial in the experimental facilities of Feeds and Foods Nutrition Research Center at Pukyong National University, Busan, Rep. of Korea, the external health condition of the experimental fish was immediately checked by eye observation on arrival and then the fish were not fed for 24 h. The fish were fed the control diet for two weeks to adapt to the experimental environment as well as having ongoing health status checks [42]. During the acclimatization time, the fish were found to be apparently healthy, and no external lesion or hemorrhagic gill was seen on visual observation. A total of 525 fish with an average initial weight of 12.1 ± 0.04 g (mean ± SD) were arbitrarily transferred (25 fish/40 L tank) into each of the 21 glass water tanks which received a continuous flow of filtered seawater (1.2 L/min). Among the 21 tanks for the 7 dietary treatments, each experimental diet was assigned to three replicate tanks (1 treatment in triplicates) with a completely randomized design. Saturated aeration (near 10 ppm) was supplied using an aerator in water over the experimental period to protect the fish from suffocation, and a constant water temperature was maintained using thermostats. The water pH was regularly monitored during the experiment. The water temperature and pH were recorded to be 19.0 ± 1.0 °C and 7.5 ± 0.3, respectively. Fish feeding was done twice a day (09:00 and 19:00 h) for 8 weeks at the satiation rate of 2~3% wet body weight of the fish per day. Moreover, fish feeding was adjusted based on the number and weight of the fish that died after immediate discarding the dead fish from the rearing tank. The fish rearing tanks were regularly siphoned to remove unfed feeds after 1 h of fish feeding and the tanks were always kept clean by rubbing with scrubber to remove algae or fungal attachments that were inside the tanks.

### 2.5. Sample Collection and Analyses

At the end of the 8 weeks of feeding trials, the fish were unfed for 24 h, and the total number and weight of the fish in each tank were measured to calculate final weight (FW), weight gain (WG), specific growth rate (SGR), feed efficiency (FE), protein efficiency ratio (PER), and the survival rate (SUR). For the histological and somatic index analyses, five fish from each tank were separated and the individual weights of the fish were recorded. The fish were then dissected to collect liver and visceral weight for the estimation of hepatosomatic index (HSI) and viscerosomatic index (VSI). Afterwards, the intestine from the same fish were used for histological observation and measurements. In addition, three fish from each tank were separated to determine the whole-body proximate chemical composition of the fish. Furthermore, three fish per tank were additionally collected for serum biochemical and non-specific immune parameter analyses. For this, the fish were euthanized with ethylene glycol phenyl ether (200 mg/L for 5–10 min) and then blood was obtained by the puncturing of the caudal vein near the lateral line on skin with 1-mL disposable syringe without anticoagulant. Finally, the serum was isolated from the whole blood samples by centrifuging the blood at 5000× *g* for 10 min and the supernatant liquid part (serum) was stored at −70 °C for further analysis of serum biochemical parameters such as glutamic pyruvic transaminase (GPT), glutamic oxaloacetic transaminase (GOT), glucose, and total protein (TP) and non-specific immune-related enzymes such as lysozyme and myeloperoxidase (MPO) activities. 

### 2.6. Proximate Composition Fish and Feeds

Proximate composition analyses of the experimental diet and fish body were determined by the standard methods of AOAC [43]. Samples of the diets and fresh fish were dried at 105 °C to a constant weight to determine their moisture contents. The crude ash content was determined by incineration of the samples at 550 °C. The crude protein was measured using the Kjeldahl method (N × 6.25) after the determination of nitrogen (N) content through acid digestion, distillation, and titration of the samples. Finally, the crude lipid content of sample was determined by ethyl-ether extraction method using a Soxhlet apparatus 1046 (Tacator AB, Hoganas, Sweden). 

### 2.7. Serum Biochemical Analysis

The serum GPT, GOT, glucose, and TP levels of fish (0.1 mL for each sample) were measured by using kits (Fuji Photo Film Ltd., Tokyo, Japan) with a chemical analyzer (Fuji DRI-CHEM 3500i, Fuji Photo Film Ltd., Tokyo, Japan) following the manufacture’s instruction.

### 2.8. Serum Enzyme Activity Analysis

Serum lysozyme activity (EC 3.2.1.17) was analyzed by a turbidometric assay, as reported by Hultmark et al. [44]. Briefly, the serum samples (0.2 mL each) according to the dietary treatment were added to bacterial suspension, *Micrococcus lysodeikticus* (0.75 mg/mL) with 0.1 M sodium phosphate buffer (pH 6.4). According to the dietary treatments, the reactions were carried out in a 96-well microplate at 20 °C and an absorbance at 570 nm between 0 and 30 min using a microplate reader (TECAN M200 Plate Reader, Tecan Trading AG, Männedorf, Switzerland). A reduction in the absorbance of 0.001/min was regarded as one unit of lysozyme activity. The lysozyme activity was measured using the following equation:

Units/mL = (∆A_570_/minutes × 1000)/mL enzyme in reaction mixture

Serum myeloperoxidase (MPO) activity (EC 1.11.1.7) was measured based on Quade and Roth [45] with minor modifications. Briefly, 20 µL serum samples from olive flounder fish was diluted with 80 µL HBSS (Hanks Balanced Salt Solution, Sigma-Aldrich, St. Louis, MO, USA) without calcium ion (Ca^2+^) or magnesium ion (Mg^2+^) in a 96-well microplate. Afterwards, 35 µL of 20 mM TMB (3,3′,5,5’ tetramethylbenzidine hydrochloride, Sigma-Aldrich, St. Louis, MO, USA) was added to the reaction mixture and then 35 μL of 5 mM H_2_O_2_ (hydrogen peroxide) was added to the wells of the microplate. The color change reaction was acquired during incubation time that lasted for 2 min and then 35 µL of 4M H_2_SO_4_ (sulfuric acid) was added in the mixture. Finally, the absorbance of the serum samples was measured at 450 nm in a microplate reader (TECAN M200 Plate Reader, Tecan Trading AG, Männedorf, Switzerland) based on the dietary treatments.

### 2.9. Quantitative Reverse Transcriptase Polymerase Chain Reaction (qRT-PCR) Analysis

The tissue fragments of head kidney (HK) that were collected from the dissected fish were immediately preserved at −80 °C in TRIzol reagent (Thermo Fisher Scientific, San Jose, CA, USA) for RNA extraction. Total RNA was extracted from 0.5 g of olive flounder tissue using TRIzol Reagent (Thermo Fisher Scientific, San Jose, CA, USA). Moreover, fish tissues were analyzed after quantitative analysis and purity assessment using a microvolume UV-Vis spectrophotometer (NanoDrop One, Thermo Fisher Scientific, San Jose, CA, USA). Then, the DNase I enzyme (Cosmogenetech, Seoul, Rep. of Korea) was mixed with the isolated RNA from tissues to exclude genomic DNA from the samples. Afterwards, we used the M-MuLV reverse transcriptase (Cosmogenetech) to produce complementary DNA (cDNA) from the samples. The qRT-PCR analyzer (Bio-Rad CFX96, Bio-Rad, Hercules, CA, USA) was then run with SYBR-Green reagent to determine the expression levels of the four selected immune-related genes such as *FGH* (flounder growth hormone), *IL-1β* (interleukin 1β), *IL-10* (Interleukin 10), and *β-actin* (beta-actin as house-keeping gene) which were calculated by using CFX software in triplicates (CFX manager software version 2.0, Bio-Rad) (Table 2).

### 2.10. Histology

A histological study was carried out using the standard protocol [17]. Briefly, the anterior intestine from the dissected fish of each treatment (*n* = 5 per tank) was fixed in the 10% neutral buffered formalin immediately after the collection of the samples. Furthermore, cleaning, infiltration, and dehydration processes were executed using a series of alcohol applications in increasing concentrations. The tissue blocks were sectioned (5 μm thickness) using a rotary microtome machine (HistoCore, Leica Biosystems, Buffalo Grove, IL, USA). Finally, the tissue sections were stained routinely with hematoxylin and eosin (H&E) as per schedule and mounted with Canada balsam (mountant). The mounted tissue sections were observed under a compound light microscope (AX70 Olympus, Tokyo, Japan) that was adjusted with a digital camera (DIXI Optics, Daejeon, Rep.of Korea). The images of the histological slides were analyzed by using an image software (Image J 1.32j, National Institute of Health, Bathesda, MD, USA). The statistical analysis of the villus height data used a pool of at least 6 images.

### 2.11. Challenge Test against Edwardsiella tarda

For the challenge test experiment, we collected the pathogenic bacteria, *Edwardsiella tarda* (ATCC 15947, American Type Culture Collection, Manassas, VA, USA) from the Department of Biotechnology, Pukyong National University, Busan, Rep. of Korea. The origin of the collected *E. tarda* bacteria was diseased brook flounder and the pathogenic bacteria was reproduced on TSA agar (tryptic soy agar, Sigma-Aldrich, USA) plates at 26 °C for 24 h in laboratory condition for utilization in the challenge test. The reason behind the challenge test with *Edwardsiella tarda* is that olive flounder fish are particularly susceptible to the bacteria and it is the causal agent of the septicemic disease, Edwardsiellosis. Therefore, at the end of the final sampling of the feeding trial, seven fish from each tank were redistributed in the tanks based on the previous dietary treatments. However, in the challenge test experiment, water inside each experimental tank was in a non-circulating condition and no water was exchanged during the challenge test program. In all the tanks, the fish were intraperitoneally injected (i.p.) with 100 μL of *E. tarda* (2 × 10^7^ CFU/mL) solution per fish to spread the bacteria in peritoneal cavity and quickly diffuse the bacterial solution in the tissue of gastrointestinal tract of fish, which is a widely used injecting method for in vivo challenge test study in fish [17,18,19,20,24,25,27,34,37,40]. The mortality of the fish in each tank was monitored and recorded daily (if any) up to 15 days. Furthermore, immediately after the collection of the dead fish, swabs from skin, gill, liver, and kidney of the dead fish was checked on modified selective agar for the confirmation of pathogenicity of *E. tarda*. After the bacterial culture procedure with swabs and the observation of black spots on the skin of dead fish, we confirmed that the mortality of fish was due to the infection of *E. tarda* bacteria.

### 2.12. Statistical Analysis

The fish tank mean values (*n* = 3) were used for statistical analysis. Normality and homogeneity of variance were assessed for all data using the Shapiro–Wilk and O’Brien tests, respectively. All data were analyzed by one-way analysis of variance (ANOVA) to test for the effects of the dietary treatments. A Fisher’s protected least significant difference (LSD) post hoc test was used to compare the means amongst the treatments with significant effects. The treatment effects were considered with the significant level at *p* < 0.05. All statistical analyses were tested using SAS version 9.1 analytical software (SAS Institute, Cary, NC, USA). A survivability curve for the challenge test was based on Kaplan and Meier [46] to determine the differences in survival rate among the treatment groups.

## 3. Results

### 3.1. Growth Performance of Fish

The growth performance of juvenile olive flounder that were fed the different experimental diets for eight weeks is presented in Table 3. At the end of the feeding trial, FBW, WG, and SGR of fish that were fed the BSSJ10, BSWB60, and EFSH30 diets were significantly higher than those of fish fed the CON, OTC, and AMO diets (*p* < 0.05). However, there were no significant differences in FBW, WG, and SGR of fish fed the BSSJ10, BSWB60, EFSH30, and GRO diets. The FE and PER of fish that were fed the GRO diet was significantly higher than those of fish that were fed the CON diet (*p* < 0.05). However, fish that were fed the probiotic-supplemented diets did not show significant differences in FE and PER compared to those of fish fed the GRO diet (*p* > 0.05). Moreover, fish that were fed the experimental diets were not significantly differed in terms of SUR, CF, his, and VSI (*p* > 0.05).

### 3.2. Whole-Body Proximate Composition

The whole body proximate composition of fish that were fed the experimental diets is presented in Table 4. The results showed that there were no significant differences in the crude protein, crude lipid, moisture, and crude ash contents among fish that were fed the different diets for eight weeks.

### 3.3. Serum Biochemical Parameters of Fish Blood

The serum biochemical composition of juvenile olive flounder that were fed the experimental diets is presented in Table 5. There were no significant differences in the GOT, GPT, glucose, and total protein (TP) contents among the fish that were fed the experimental diets for eight weeks.

### 3.4. Immune Enzymes Analyses in Fish

**Figure 1:**
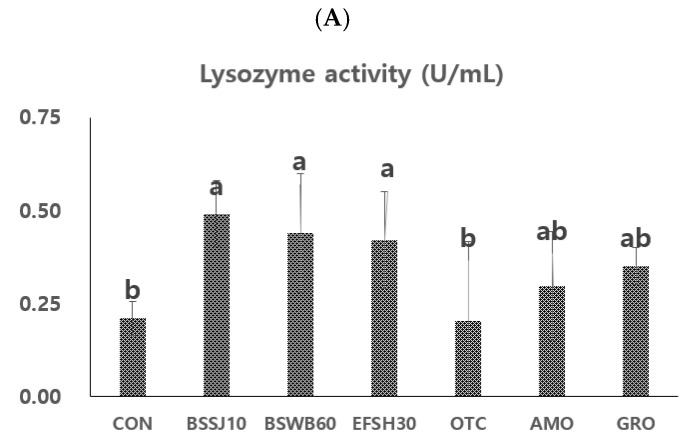

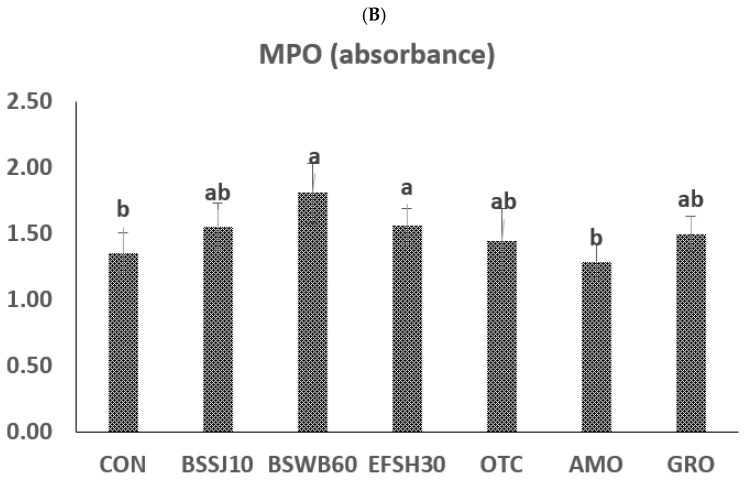
Lysozyme (**A**) and myeloperoxidase (MPO) activities (**B**) of juvenile olive flounder that were fed the seven experimental diets for eight weeks. Diets denotes, CON = control or basal diet; BSSJ10 = *B. subtilis* SJ-10 at 1 × 10^8^ CFU/kg diet; BSWB60 = *B. subtilis* WB60 at 1 × 10^8^ CFU/kg diet; EFSH30 = *E. faecium* SH30 1 × 10^7^ CFU/kg diet; GRO = processed yeast, GroPro Aqua at 0.35 g/kg diet; OTC = oxytetracycline at 4 g/kg diet and AMO = amoxicillin at 4 g/kg diet. Each value represents the mean ± SEM (*n* = 3). Different letters (a, b) are significantly (*p* < 0.05) different by LSD test.

The innate immune enzyme activities of juvenile olive flounder that were fed the different experimental diets for eight weeks are shown in Figure 1A,B). The lysozyme activity of fish that were fed the BSSJ10, BSWB60, and EFSH30 diets were significantly higher than those of fish that were fed the CON and OTC diets (*p* < 0.05). However, there were no significant differences in lysozyme activities of fish that were fed the BSSJ10, BSWB60, EFSH30, AMO, and GRO diets (*p* > 0.05). Furthermore, fish that were fed the CON, OTC, AMO, and GRO diets showed no significant differences in terms of lysozyme activities (Figure 1A).

In the case of the MPO activities (Figure 1B), fish that were fed the BSWB60 and EFSH30 diets showed significantly higher MPO activity than those of fish that were fed the CON and AMO diets (*p* < 0.05). However, there were no significant differences in MPO activities among the groups of fish that were fed the BSSJ10, BSWB60, EFSH30, AMO, and OTC diets as well as the fish fed the CON, BSSJ10, GRO, OTC, and AMO diets (*p* > 0.05).

### 3.5. Immune and Growth Related Gene Expressions in Fish

**Figure 2:**
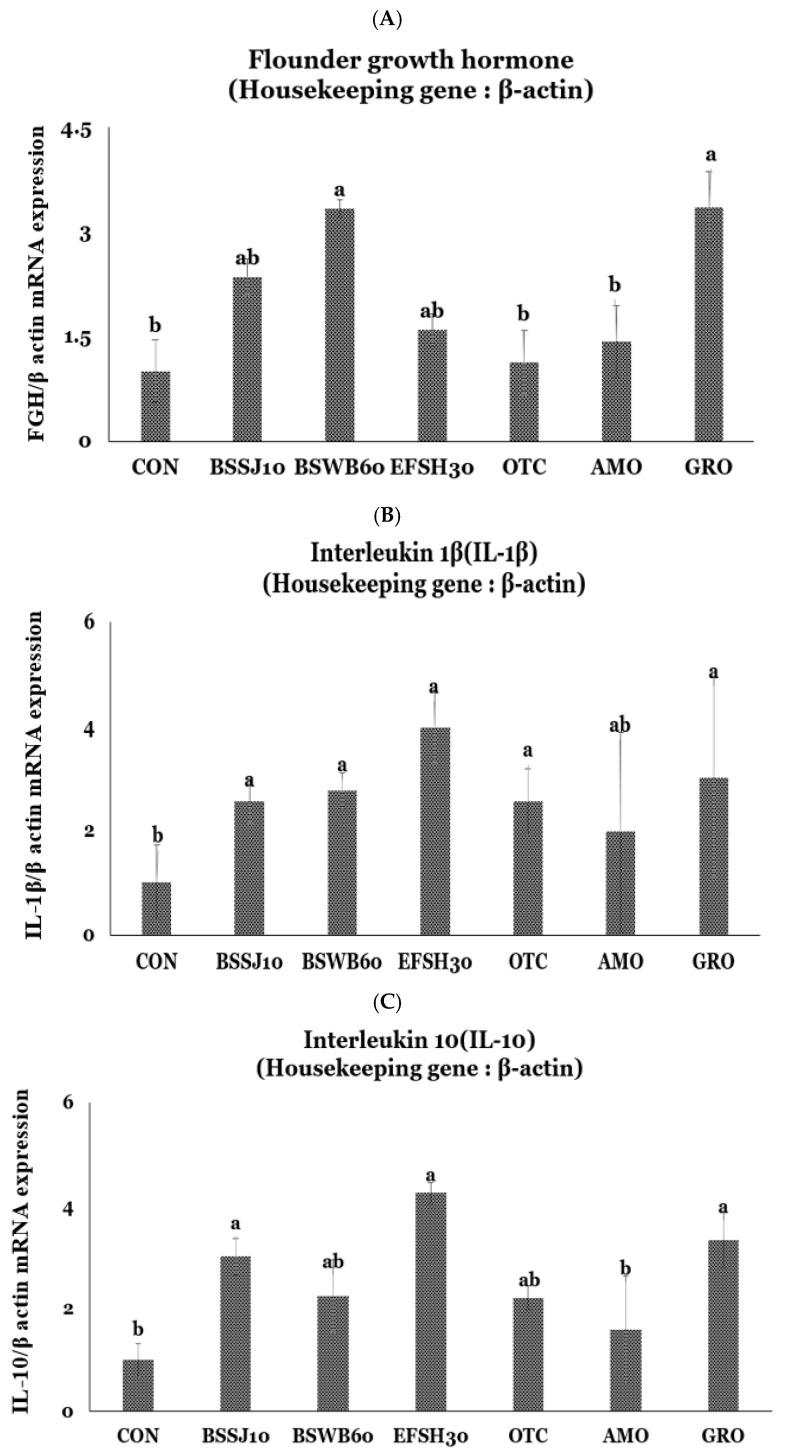
Relative expression levels of flounder growth hormone, FGH (**A**); interleukin-1β, IL-1β (**B**); and interleukin-10, IL-10 (**C**) mRNA of head kidney from juvenile olive flounder fed the seven experimental diets for eight weeks. Diets denotes, CON = control or basal diet; BSSJ10 = *B. subtilis* SJ-10 at 1 × 10^8^ CFU/kg diet; BSWB60 = *B. subtilis* WB60 at 1 × 10^8^ CFU/kg diet; EFSH30 = *E. faecium* SH30 1 × 10^7^ CFU/kg diet; GRO = processed yeast, GroPro Aqua at 0.35 g/kg diet; OTC = oxytetracycline at 4 g/kg diet and AMO = amoxicillin at 4 g/kg diet. Each value represents the mean ± SEM (*n* = 3). Different letters (a, b) on the bars are significantly (*p* < 0.05) different by LSD test.

Fish immune and growth-related gene expressions such as FGH, IL-1β, and IL-10 in relation to the housekeeping gene β-actin in the head kidney (HK) of juvenile olive flounder are presented in Figure 2A–C. The results showed that FGH gene expression of fish (Figure 2A) that were fed the BSWB60 and GRO diets were significantly higher than those of fish that were fed the CON, OTC, and AMO diets (*p* ˂ 0.05). However, there were no significant differences in the FGH gene expression of fish that were fed the BSSJ10, BSWB60, EFSH30, and GRO as well as the fish that were fed the CON, BSSJ10, EFSH30, OTC, and AMO diets (*p* > 0.05). In addition, IL-1β gene expression of fish (Figure 2B) that were fed the BSSJ10, BSWB60, EFSH30, OTC, and GRO diets were significantly higher than those of the fish that were fed the CON diet (*p* < 0.05). However, there were no significant differences in IL-1β gene expression of the fish that were fed the CON and AMO diets as well as the fish that were fed the BSSJ10, BSWB60, EFSH30, OTC, AMO, and GRO diets (*p* > 0.05). The interleukin 10 (IL-10) gene expression of fish (Figure 2C) that were fed the BSSJ10, EFSH30, and GRO diets were significantly higher than those of the fish that were fed the CON and AMO diets (*p* ˂ 0.05). However, there were no significant differences in IL-10 gene expression of fish that were fed the BSSJ10, BSWB60, EFSH30, OTC, and GRO diets as well as the fish that were fed the CON, BSWB60, OTC, and AMO diets (*p* > 0.05).

### 3.6. Histology of Anterior Intestine of Fish

**Figure 3:**
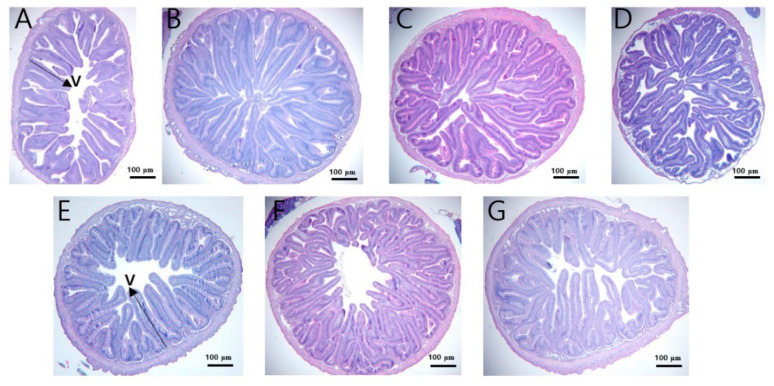
Details of the anterior intestinal histological examination of juvenile olive flounder that were fed the experimental diets for eight weeks: (**A**) CON; (**B**) BSSJ10; (**C**) BSWB60; (**D**) EFSH30; (**E**) OTC; (**F**) AMO; and (**G**) GRO diets (hematoxylin and eosin staining; scale bar = 100 μm; Original magnification × 100).

**Figure 4:**
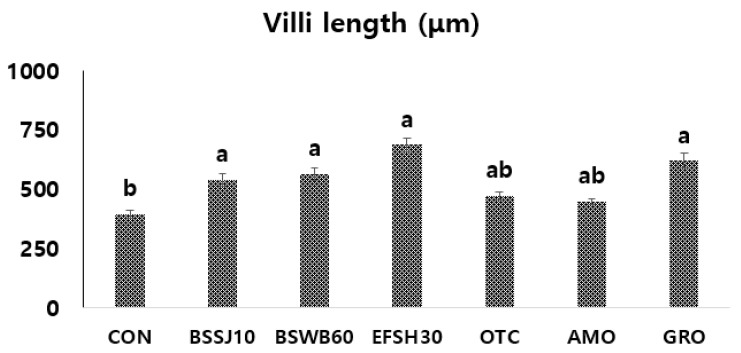
Villi length from the anterior intestine of juvenile olive flounder that were fed the experimental diets for eight weeks. Diets denotes, CON = control or basal diet; BSSJ10 = *B. subtilis* SJ-10 at 1 × 10^8^ CFU/kg diet; BSWB60 = *B. subtilis* WB60 at 1 × 10^8^ CFU/kg diet; EFSH30 = *E. faecium* SH30 1 × 10^7^ CFU/kg diet; GRO = processed yeast, GroPro Aqua at 0.35 g/kg diet; OTC = oxytetracycline at 4 g/kg diet and AMO = amoxicillin at 4 g/kg diet. Each value represents the mean ± SEM (*n* = 3). Different letters (a, b) on the bars are significantly (*p* < 0.05) different by least significant difference (LSD) test.

The histological analysis and statistical significance in the villi length of the anterior intestine of olive flounder that were fed the different experimental diets for eight weeks are shown in Figure 3 and Figure 4. Fish in the groups of BSSJ10, BSWB60, EFSH30, and GRO diets clearly exhibited better intestinal histomorphology with an increased size of villus compared to the CON diet. In addition, fish that were fed the probiotics (BSSJ10, BSWB60, and EFSH30) and the processed yeast-supplemented (GRO) diets had better intestinal villi arrangement compared to the fish that were fed the antibiotics (OTC and AMO)-supplemented diets (Figure 3). Likewise, the statistical data showed that the intestinal villi length of fish that were fed the BSSJ10, BSWB60, EFSH30, and GRO diets were significantly higher than those of fish that were fed the CON diet. However, there were no significant differences in the villi length of fish fed the CON, AMO, and OTC diets (Figure 4).

### 3.7. Challenge Test against Edwardsiella tarda

**Figure 5:**
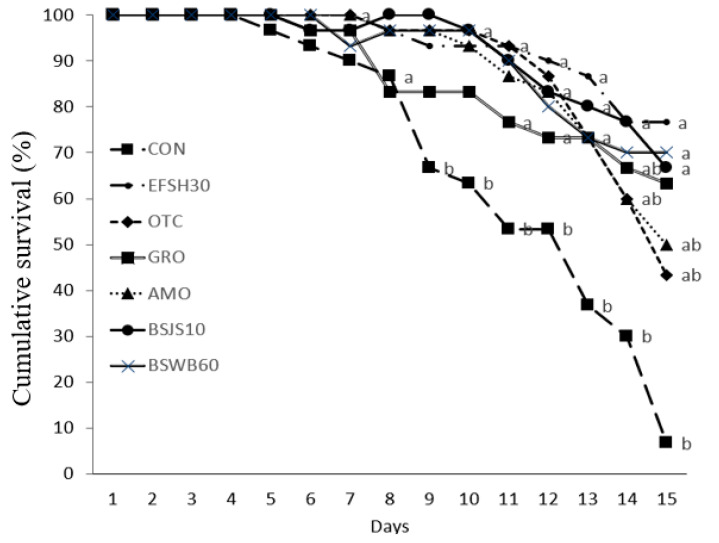
The cumulative survival rate after challenge with *Edwardsiella tarda* for 15 days in juvenile olive flounder that were fed the seven experimental diets for eight weeks. Each value represents the mean ± SEM (*n* = 3). Different letters (a, b) are significantly (*p* < 0.05) different by LSD test.

The cumulative survival rate of juvenile olive flounder that were tested with *E. tarda* for 15 days is shown in Figure 5. During the challenge test with *E. tarda*, the first mortality in fish was observed on the 4th day and it was significantly pronounced after the 8th day of injection. After the 15 days of the challenge test, the cumulative survival rate of fish that were fed the BSSJ10, BSWB60, EFSH30, and GRO diets were significantly higher than those of fish that were fed the CON diet (*p* < 0.05). However, there were no significant differences in the cumulative survival rate of fish that were fed the AMO, OTC, and CON diets (*p* > 0.05).

## 4. Discussion

### 4.1. Growth and Somatic Indices of Olive Flounder

The results of the present study clearly show the efficiency of bacterial probiotics and processed yeast on the growth performance parameters of olive flounder. Our study demonstrated that dietary probiotic bacteria had significantly higher growth increments in juvenile olive flounder than the control and antibiotics that were supplemented in the diets. Moreover, fish that were fed the processed yeast-supplemented diet had no significant differences in WG compared to the control- and antibiotics-supplemented diets. The results might be due to the higher digestion and assimilation of probiotic bacteria-supplemented diets in fish than those of the control and the antibiotics-supplemented diets. There have been a significant number of studies that have reported the beneficial effects of probiotic bacteria group such as *Bacillus*, *Vibrio*, and *Lactobacillus* spp. which are extensively used in commercial aquaculture [17,18,19,20,22,25,26,40,47,48,49]. The beneficial effects of probiotic bacteria *Bacillus subtilis* WB60 is reported in our recent studies in the freshwater Japanese eel [17]. The addition of probiotics in feed in fish has been reported to increase the digestion rate of fish and shrimp by the activity of digestive enzymes such as protease, amylase, and lipase [50,51,52], improving the bioavailability of feed [48]. Also, *B. Subtilis* has been reported to promote nutrient absorption in the intestines by improving the activity of several enzymes [53]. In agreement of the present study, similar results were reported in the studies on tilapia [54], silver barb [55], white shrimp [47], and sea cucumber [53]. In this study, the results show that the feed efficiency of processed yeast (GRO) was significantly higher than those of the control diet. The beneficial effects of yeast on the growth, feed utilization, gut health, and the first line of defense has been recently reported in juvenile barramundi by Siddik et al. [56] which is in line with the present study. The hepatosomatic index (HSI), viscerosomatic index (VSI), and condition factor (CF) are important tools to assess the physiological and biological conditions of fish in terms of liver, viscera, and the health index of fish. In the present study, there were no significant differences that were found in the somatic indices such as the HSI, VSI, and CF which indicate that liver, viscera, and fish health status was not affected by the dietary supplementation of probiotic bacteria and yeast. Likewise, the whole body proximate composition of fish such as crude protein, crude lipid, crude ash, and moisture in fish that were fed the experimental diets support the results that were reported in Japanese eel and whiteleg shrimp [17,26]. The results of the growth and feed utilization data postulated that the three dietary strains of probiotic bacteria and the processed yeast can be used as alternatives to antibiotics in the diets of juvenile olive flounder, which might be achieved through the reduction of pathogenic and growth of beneficial bacteria, adherence of probiotic bacteria to mucosa and epithelium, as well as strengthening the gut barrier function of GIT in fish [57,58].

### 4.2. Hematology of Olive Flounder

Serum biochemical composition is an important tool to figure out the health condition of fish and shrimp [59]. In this study, the serum GPT, GOT, GLU, and TP levels were not significantly changed by the dietary treatments, which may demonstrate that the dietary supplementation of probiotics, yeast, or antibiotics has equal or no effects on health status in terms of liver disorders (GPT and GOT) or physiological stress (GLU and TP) of juvenile olive flounder. This is in accordance with the results that reported that there were no effects of dietary single or multi-probiotics on the hematological parameters of starry flounder, rainbow trout, whiteleg shrimp and barramundi [24,26,56,60].

### 4.3. Non-Specific Immune Responses and Growth Hormone in Olive Flounder

The results of the non-specific immune responses of the present study demonstrated the positive effect of dietary probiotics in olive flounder. The innate or non-specific immune response is considered as the fundamental and first line of defense tool for fish [61]. Lysozyme activity is an important humoral immune enzyme that has bactericidal properties in terms of hydrolysis of the peptidoglycan layer of the bacterial cell wall [21]. The lysozyme enzyme can also activate the complement system and helps in phagocytosis through the opsonisation process in association with leukocytes in the animal body [60,62]. In addition to the bactericidal or bacteriolytic activity against Gram-positive or Gram-negative bacteria, the lysozyme enzyme can also show its anti-inflammatory and antiviral activities in animals [63]. Furthermore, the lysozyme enzyme can promote growth in animals [64] which is evidenced in the present study with the concomitant increment of growth and lysozyme activity in olive flounder after the supplementation of dietary probiotic bacteria and processed yeast in fish. On the other hand, myeloperoxidase (MPO) is also an important heme-peroxidase enzyme that is mainly present in neutrophils and, to a lesser degree, in monocytes of the innate immune system [65]. Myeloperoxidase can show its radical scavenging activity through regulating the reactive oxygen species (ROS) and has ability to kill bacteria and other invading pathogens through the production of hypochlorous acid (HOCl) and strong oxidants [66,67]. In the present study, the lysozyme and MPO activities in fish were significantly increased with the dietary supplementation of probiotic bacteria compared to the control diet, which is in agreement with the results that were reported in Japanese eel [17]. In contrast to the present study, there were no significant differences in MPO activities that were reported for starry flounder, rainbow trout and whiteleg shrimp that were fed the probiotics-supplemented diets compared to the control diet [21,26,60]. Moreover, in this study, there were no significant differences in lysozyme and MPO activities in fish that were fed probiotics- or processed yeast (GroPro-Aqua)-supplemented diets than the fish that were fed an antibiotics-supplemented diet. This might be attributed to the usefulness of probiotics or yeast as the alternatives of antibiotics in the diet of juvenile olive flounder based on innate immune enzyme activities in fish.

Gene expressions in fish, such as the growth- and cytokine-related mRNA expressions, may provide information on the actual changes in growth and innate immunity as well as the adaptive immune functions in fish on a genetic level. In this study, we evaluated the dietary probiotics, yeast, and antibiotics based on flounder growth hormone (*FGH*) as well as cytokines, *IL-1β* and *IL-10* gene expressions in relation to the housekeeping gene, *β-actin*. Our results showed that dietary probiotics (BSWB60) and processed yeast (GRO) had significantly a higher influence on *FGH* than did the CON and antibiotic (OTC and AMO)-supplemented diets which indicate the beneficial effect of probiotics and yeast over CON and antibiotic-supplemented diets in terms of growth performance in olive flounder, which is in agreement with Back et al. [16]. Inflammation is a rapid response of the innate immune system where cells that are engaging in inflammation undergo drastic changes of their transcriptomes and can cause trauma or tissue damage [68]. There are three families of signal-regulated transcription factors (SRTFs) that are involved in inflammation such as signal transducers and activators of transcription (STATs), interferon regulatory factors (IRFs), and nuclear factor κB (NFκB) [68]. To determine the changes in gene expressions during the inflammatory process, tight and coordinate regulation of gene expression by environmental cues, microbial or danger-associated molecules or cytokines, are mandatory [68]. Cytokines are usually small protein molecules (~5–25 kDa) that can regulate the biological functions such as stress, non-specific and adaptive immune responses, the production of cellular components in blood, and the recovery of body injury, mostly through the extracellular signaling pathway on the cell cortex [25,69]. The cytokine, *IL-1β* is a major player in the immune responses of fish, as in mammals, and is involved in the modulation of autoimmune inflammation [69]. On the other hand, *IL-10* is also an anti-inflammatory cytokine that plays a vital role in immuno-regulation for inflammation [70,71]. In the present study, the cytokine genes such as *IL-1β* and *IL-10* mRNA expression levels were significantly higher in fish that were fed the probiotic bacteria-, processed yeast (GRO)-, and antibiotic-supplemented diets which indicate the higher immunomodulatory effect of the probiotic bacteria, yeast, or antibiotics in the diets of juvenile olive flounder. The mechanism of immunomodulatory effects by probiotic bacteria or yeast can be explained in terms of recognition of the toll-like receptors (TLRs) and the upregulation of anti-inflammatory cytokines and growth factors which modulate the NFκB and mitogen-activated protein kinase (MAPK) pathways [57,58]. In agreement with our study, Won et al. [25] reported the significant upregulation of *IL-1β* gene expression of fish that were fed the *B. subtilis* WB60- and OTC-supplemented diets compared to the control diet. In contrast to our study, Hasan et al. [49] did not find any significant difference in *IL-10* gene expression in different organs (kidney, liver, gill, and spleen) of fish that were fed the *B. subtilis* SJ-10-supplemented and the control diets. Furthermore, Siddik et al. [56] postulated the significantly positive effect in terms of higher *IL-10* gene expression in fish that were fed the yeast-supplemented diet compared to the control diet. 

### 4.4. Histomorphology of Intestine in Olive Flounder

The intestinal morphology parameters, especially villi length, is a reflection of a well gastrointestinal tract (GIT) in fish, which helps to absorb and assimilate nutrients in the GIT of fish. In any organisms, the intestine is an integral part of the body to transfer nutrients from gut wall into the blood circulation through the digestion and absorption of the diet. Hence, the larger surface area of the intestinal villi can absorb more nutrients in the GIT and those nutrients can then be distributed in different organs of the animal body [72,73]. Thus, the proper digestion and utilization of diets depends on the GIT health status in the animal [54,73]. In the present study, the villi length of fish that were fed the probiotic bacteria (BSSJ10, BSWB60, and EFSH30)- and the processed yeast (GRO)-supplemented diets showed significantly higher villi length compared to the control diet, which might be attributed to the better health status of GIT as well as a higher absorption of nutrients in the GIT. The results of the histomorphological data supported the growth enhancement data on fish of the present study. Interestingly, in the present study, the villi length in relation to the histomorphological evaluation also showed that fish that were fed the control diet had small, thin, and diffused villi, whereas the fish that were fed the probiotic bacteria- and the yeast-supplemented diets had elongated, highly dense, and robust villi and they were slightly better than the antibiotics-fed fish in terms of elongation of the villi. In agreement with the present study, we found similar results in fish that were fed the additives- or probiotics -supplemented diets in rainbow trout, olive flounder, silver barb, and shrimp [13,26,52,55]. Likewise, Siddik et al. [56] reported the higher villi length in oral administration of the yeast-supplemented diet than the control diet in barramundi fish. Overall, the growth and histomorphological findings corroborated that the dietary probiotic bacteria and processed yeast can replace the antibiotics in the diet of juvenile olive flounder.

### 4.5. Disease Resistance in Olive Flounder Challenged with Edwardsiella tarda

In this study, after the 15-day challenge test, we observed significantly higher disease resistance against *Edwardsiella tarda* in terms of the cumulative survival rate in fish that were fed the probiotic bacteria and the processed yeast-supplemented diets than in the group of fish that were fed the control diet, which suggests the enhancement of disease resistance in fish due to the supplementation of the additives in the diet. However, we could not find significant differences but a numerically higher survival rate in fish that were fed the probiotic bacteria- and yeast-supplemented diets than in fish that were fed the antibiotics-supplemented diet. This indicates the potential uses of probiotic bacteria (BSSJ10, BSWB60, and EFSH30) and processed yeast (GRO) as the alternative of antibiotics in juvenile olive flounder. The mechanism can be attributed in terms of enhancing the mucosal and systemic immunity as well as the direct action of the probiotic bacteria or yeast on pathogenic bacteria through the production of inhibitory substances such as lysozymes, proteases, siderophores, hydrogen peroxide, or bacteriocins in the GIT of fish [74]. The results are in agreement with those that were previously reported in starry flounder, Japanese eel, barramundi, and shrimp [17,21,26,56].

## 5. Conclusions

Taken together, the results of our study demonstrated that dietary supplementation of probiotic bacteria such as *B. subtilis* (10^8^ CFU/g diet), *E. faecium* (10^7^ CFU/g diet), and processed yeast (GroPro-Aqua at 0.35% diet) can be used as alternatives to antibiotics without compromising the growth and health status of juvenile olive flounder. Further research is warranted to evaluate the effects of the probiotic bacteria and yeast on their supplementation with different combinations in the diets of fish and shrimp species.

## Figures and Tables

**Table 1:** The composition of the basal diet for olive flounder (% of dry matter basis).

Ingredients	Percentage (%)
Fish meal, anchovy ^1^	45
Soybean meal	12
Starch ^2^	3.8
Wheat flour	7
Blood meal	4.5
Squid liver powder	5.5
Meat and bone meal	8
Poultry by product meal	4.5
Fish oil ^3^	4.3
Vitamin premix ^4^	1.2
Mineral premix ^5^	1.2
Etc ^6^	3.0
**Proximate analysis (% of DM basis)**	
Moisture	8.56
Crude protein	56.2
Crude lipid	8.35
Crude ash	11.4

^1^ Suhyup feed Co. Uiryeong, Korea; ^2^ The feed Co. Goyang, Korea; ^3^ Jeil feed Co. Hamman, Korea; ^4^ Contains (as mg/kg in diets): Ascorbic acid, 300; dl-Calcium pantothenate, 150; Choline bitate, 3000; Inositol, 150; Menadion, 6; Niacin, 150; Pyridoxine · HCl, 15; Rivoflavin, 30; Thiamine mononitrate, 15; dl-α-Tocopherol acetate, 201; Retinyl acetate, 6; Biotin, 1.5; Folic acid, 5.4; Cobalamin, 0.06; ^5^ Contains (as mg/kg in diets): NaCl, 437.4; MgSO_4_·7H_2_O, 1379.8; ZnSO_4_·7H_2_O, 226.4; Fe-Citrate, 299; MnSO_4_, 0.016; FeSO_4_, 0.0378; CuSO_4_, 0.00033; Ca(IO)_3_, 0.0006; MgO, 0.00135; NaSeO_3_, 0.00025; ^6^ Calcium phosphate, Lecithin, Betaine, Taurine, Choline, Vitamin C, Lysine, Methionine.

**Table 2:** Primers used for real-time PCR analysis ^1^.

Gene	Sense	Primer Sequence (5′ → 3′)	Size (bp)	Accession No.
*β-actin*	F	CAGCATCATGAAGTGTGACGTG	107	HQ386788.1
	R	CTTCTGCATACGGTCAGCAATG		
*FGH*	F	CGCCGTATGGAAACTCTGAACT	160	M23439.1
	R	GGGTGCAGTTAGCTTCTGGAAA		
*IL-1β*	F	ATGGAATCCAAGATGGAATGC	250	KF025662.1
	R	GAGACGAGCTTCTCTCACAC		
*IL-10*	F	AGCGAACGATGACCTAGACACG	114	KF025662.1
	R	ACCGTGCTCAGGTAGAAGTCCA		

^1^ *FGH*: flounder growth hormone; *IL-1β*: Interleukin 1β; *IL-10*: Interleukin 10.

**Table 3:** Growth performance of olive flounder that were fed the experimental diets for eight weeks ^1^.

	Diets							Pooled SEM ^12^
	CON	BSWB60	BSSJ10	EFSH30	GRO	OTC	AMO	
IBW ^2^	12.5 ^ns^	12.4	12.6	12.2	12.3	12.2	12.4	0.39
FBW ^3^	35.6 ^b^	42.5 ^a^	41.3 ^a^	41.5 ^a^	40.0 ^ab^	37.5 ^b^	36.9 ^b^	0.39
WG (%) ^4^	185 ^b^	238 ^a^	233 ^a^	241 ^a^	223 ^ab^	205 ^b^	201 ^b^	7.04
SGR (%/day) ^5^	1.90 ^b^	2.21 ^a^	2.18 ^a^	2.23 ^a^	2.13 ^ab^	2.02 ^b^	2.00 ^b^	0.32
FE (%) ^6^	174 ^b^	178 ^ab^	174 ^ab^	174 ^ab^	190 ^a^	181 ^ab^	183 ^ab^	14.7
PER ^7^	1.06 ^b^	1.16 ^ab^	1.14 ^ab^	1.17 ^ab^	1.15 ^a^	1.14 ^ab^	1.12 ^ab^	0.09
Survival (%) ^8^	90.0 ^ns^	91.7	90.7	91.0	95.3	91.0	93.7	5.03
HSI (%) ^9^	1.24 ^ns^	1.15	1.36	1.94	1.70	1.33	1.22	0.08
VSI (%) ^10^	2.05 ^ns^	1.74	1.94	1.94	1.24	1.88	1.74	0.05
CF ^11^	0.91 ^ns^	0.93	0.97	0.92	0.95	0.91	0.92	0.01

^1^ Values are means from triplicate groups of fish where the values in each row with different superscripts (a, b) are significantly different (*p* < 0.05); ns = non-significant; ^2^ Initial body weight; ^3^ Final body weight; ^4^ Weight gain (WG, %) = [(final wt. − initial wt.) × 100]/initial wt; ^5^ Feed efficiency (FE, %) = (wet weight gain/dry feed intake) × 100; ^6^ Specific growth rate (SGR, %) = [(log_e_ final wt. − log_e_ initial wt.) × 100]/days; ^7^ Protein efficiency ratio (PER) = (wet weight gain/protein intake); ^8^ Hepatosomatic index (HSI) = (liver wt. × 100)/body wt; ^9^ Viscerosomatic index (VSI, %) = (viscera wt. × 100)/body wt; ^10^ Condition factor = (wet weight/total length^3^) × 100; ^11^ Survival rate = [(total fish – dead fish) × 100]/total fish; ^12^ Pooled standard error of mean.

**Table 4:** Whole-body proximate composition of olive flounder that were fed the seven experimental diets for eight weeks ^1^.

	Diets							Pooled SEM
	CON	BSWB60	BSSJ10	EFSH30	GRO	OTC	AMO	
Moisture	75.1 ^ns^	75.8	72.9	75.8	76.2	76.8	76.2	0.48
Crude protein	20.3 ^ns^	20.8	21.3	21.4	20.7	22.3	20.7	0.25
Crude lipid	2.42 ^ns^	2.30	2.39	2.34	2.41	2.41	2.41	0.02
Crude ash	4.09 ^ns^	4.21	4.25	4.16	4.19	4.14	4.19	0.02

^1^ Values are means from triplicate groups of fish where the values in each row with no superscripts are non-significantly (ns) different (*p* > 0.05).

**Table 5:** Serum biochemical parameters of olive flounder that were fed the experimental diets for eight weeks ^1^.

		Diets						Pooled SEM
	CON	BSWB60	BSSJ10	EFSH30	GRO	OTC	AMO	
GOT ^2^	5.67 ^ns^	5.10	5.27	5.12	5.19	5.19	5.10	0.36
GPT ^3^	20.3 ^ns^	19.5	21.4	22.0	22.5	21.0	20.5	1.25
GLU ^4^	13.3 ^ns^	13.0	11.3	14.2	12.5	12.5	11.1	1.04
TP ^5^	3.1 ^ns^	3.3	3.4	3.2	3.4	3.3	3.5	0.06

^1^ Values are means from triplicate groups of fish where the values in each row with no superscripts are non-significantly (ns) different (*p* > 0.05) ^2^ Glutamic pyruvic transaminase (U/l) ^3^ Glutamic oxaloacetic transaminase (U/L) ^4^ Glucose (mg/dL) ^5^ Total protein (g/dL).

## Data Availability

All data is reported in this article.
